# A sector-wide response to national policy on client-centred care and support: a document analysis of the development of a range of instruments to assess clients’ experiences in the care and support for people with (intellectual) disabilities

**DOI:** 10.1186/s12913-021-07341-z

**Published:** 2021-12-04

**Authors:** Petri J. C. M. Embregts, Kees Ahaus, Mirella Minkman, Henk Nies, Pauline Meurs

**Affiliations:** 1 Tranzo, Tilburg School of Social and Behavioral Sciences, Tilburg University, Postbus 90153, 5000 LE Tilburg, The Netherlands; 2 Erasmus School of Health Policy & Management, Erasmus University Rotterdam, Rotterdam, The Netherlands; 3 Tilburg University/TIAS Business school, Tilburg, The Netherlands; 4 Vilans, national Center of Expertise for Long term Care in The Netherlands, Utrecht, The Netherlands; 5 Vrije Universiteit, Department of Organization Sciences, Amsterdam, The Netherlands

**Keywords:** Client-centred care and support, Quality of care, Quality of life, Intellectual disability, Long-term care, Assessment, CQ-index, National policy

## Abstract

**Background:**

Client-centred care serves as the foundation for healthcare policy. Indeed, various instruments for assessing clients’ experiences of care and support are increasingly used to provide insights into the quality, and client-centred nature, of the care and support provided, which, in turn, aids the development of subsequent improvements. The unique characteristics of care and support for people with intellectual disabilities (ID), such as the need for both lifelong and life-wide care and support across all aspects of clients’ lives, led to an initiative within Dutch ID care to jointly develop a range of instruments to assess the experiences of clients receiving ID care and support. Individual clients’ experiences and suggestions for improvement, which are embedded in clients’ care plan cycles, constitute the foundation of this Range of Instruments. This paper provides a unique, bottom-up, exhaustive account of the process of developing the Range of instruments used to assess the experiences of clients in the field of Dutch ID care.

**Methods:**

Relevant documents at three levels (i.e. 1) national documents, such as policy papers and governmental reports, 2) documents and reports from the Dutch Association of Healthcare Providers for People with Disabilities (VGN) along with minutes from the meetings of the expert Committee who assessed the instruments, and 3) correspondence between the Committee and developers as well as the forms used in the assessment process for each instrument) were qualitatively analysed by two researchers who had no affiliation with the development of the Range of instruments used to assess clients’ experiences in ID care and support. All of the documents were inductively coded using a thematic analytical approach. Informants who were either currently or previously involved in the development of these instruments were asked to provide clarification over the documents themselves and to explain the context in which they were produced.

**Results:**

The development of the range of instruments can be classified into four phases, namely: 1) supporting the bottom-up development of initiatives to assess clients’ experiences, 2) focusing on learning and further development, 3) stimulating exchange between the developers and users of the instruments and the Committee responsible for assessing them, and 4) further development in response to the changing times and new landscape.

**Conclusions:**

The range of instruments were found to be appropriate for a variety of clients in ID care and support, specifically in terms of assessing their individual experiences and gaining insight into their suggestions for improvement, and effective in terms of collaboratively improving the quality of ID care and support. In so doing, these instruments potentially provide an avenue through which clients’ experiences can be embedded in the process of ID care and support. Other specific features in the development of these instruments, namely their incremental adoption, ongoing evaluation and strong practice orientation, were also found to be suitable for other care contexts’ attempts to respond to the top-down policy objectives of client-centeredness and translating outcomes into direct care practice.

## Background

Recent decades have seen a paradigm shift in the field of long-term care away from the delivery of care measures and measuring the effectiveness of care and support towards focusing on outcomes and clients’ experiences of care and support. The term client-centred care is often used interchangeably with consumer-centred, user-centred, patient-centred or person-centred care. As a result, there is considerable heterogeneity in terms of definitions and interpretations [1]. In this paper, client-centred care is understood as a form of care and support that is respectful of, and responsive to, the preferences, needs, and values of individual clients [2], with the overall aim being to improve the quality of care and support, and, in turn, clients’ quality of life [3–5].

Client-centred care has now become an integral part of health , long-term and social care policy^1^ and, in fact, predominates within both curative and long-term care [6, 7], including social work [8], paediatric care [9], elderly care [10], mental healthcare [11] and hospital care [12]. Indeed, client-centeredness has also become crucial in the care and support for people with ID [13]. This is visible at the level of the individual client, were there has been a marked shift towards empowering people with ID and their relatives [14], as well as in (inter) national health policy. For instance, since the late 1980s, Dutch national health policy obliges care providers for people with ID to provide support based on client-centred planning [13]. Respect for autonomy and, consequently, the importance of actively involving people with ID in the decision-making process related to both their own support planning and healthcare policy more broadly, is also underscored in the United Nations Convention on the Rights of Persons with Disabilities [15].

Although client-centred care is increasingly embedded in both health policy and practice, it has proven difficult to translate this ethos successfully into daily practice [16, 17]. This is because there is a lack of organisational and/or financial support systems to help health professionals implement client-centred care [18, 19]. One way in which client-centred care is implemented in today’s healthcare systems is through the administering of instruments to assess clients’ experiences or level of satisfaction with the care they have received [20], with the outcomes functioning as both an important indicator of the quality of care and a tool for improvement [21]. More specifically, Dutch ID care organisations has also used valid instruments that assess clients’ perspectives of the quality of care and support they have received as an important tool through which to structurally improve and professionalise the care and support provided to clients with ID [22]. In the Netherlands, Consumer Quality Indexes (CQ-index, which are derived from the widely used CAHPS questionnaire), are generally used to quantitatively assess the quality of care from clients’ perspectives. This is a standardised system for assessing, analysing and reporting clients’ experiences of healthcare at both the organisational and team level. Each CQ-index consists of several domains with various indicators, such as accessibility of the healthcare organisation, experience of treatment by physician(s), and the quality of the communication. Above all, CQ-indexes focus on benchmarking and accountability, and are implemented in a top-down manner [23].

Despite its value, the consensus in the field of Dutch ID care was that the CQ-index, firstly, did not sufficiently incorporate topics with particular relevance to the long-term care sector, such as self-determination and participation [23], and secondly, failed to sufficiently address the specific challenges inherent to assessing and understanding the experiences of clients with ID, such as, among other things, recognised limitations in their verbal communication skills [24]. In addition, the explicit objective in the field of ID care is to collect data at the level of the individual client, in order to make adjustments and improvements to both the care process and the day-to-day lives of clients themselves [25]. A key feature of ID care is the provision of lifelong and life-wide care and support across all domains of client’s lives [26], with experiences of care and support and suggestions for improvement ultimately changing over the course of a person’s lifespan. The client population ranges from those who have mild ID (IQ 50/55-70) to those with profound intellectual and multiple disabilities (PIMD; IQ < 20 [27]), which results in a broad range of care and support provision, i.e., from community-based residential support to continuous support in more segregated residential facilities. Given the diversity of the client-base, the Dutch Association of Healthcare Providers for People with Disabilities (Dutch abbreviation VGN) deemed that a singular uniformed instrument for assessing clients’ experiences was simply not fit for purpose. Furthermore, they did not endorse the CQ-index’s predominant focus upon benchmarking and accountability, which were soundly endorsed by healthcare insurance providers, opting instead to collaboratively improve the quality of ID care and support.

Consequently, in line with similar assessment instruments for people with ID in other countries, such as the Personal Outcome Measures (POM [28]) in the USA and the Instrument for the Classification and Assessment of Support Needs (I-CAN [29]) in Australia, VGN proposed a way of assessing the quality of care that was better suited to clients with ID, ultimately developing a so-called ‘Range of instruments to assess clients’ experiences’ (hereafter referred to as ‘the Range of instruments’). Given the wide range of available instruments in the Netherlands, this Range of Instruments needed to comprise a set of valid instruments developed to fit the specific and diverse contexts of care organisations for people with ID in the Netherlands. The proposed Range of instruments should be suitably varied, so that care organisations have the ability to choose for themselves which valid instrument is best suited to their particular clients, employees, and organisation (including their organisational values). Using the ‘best fit’ instrument would make it easier to subsequently translate the outcomes to their care practice and to actually implement improvements in communication between all of the involved parties, in turn, enhancing the quality of ID care and support [30]. In this paper, we provide a unique bottom-up, exhaustive account of the process of developing the Range of instruments used to assess the experiences of clients in the field of Dutch ID care. We use the Netherlands and ID care as a case study into how we approached the task of making clients’ experiences explicit in a systematic manner, so that they could be used as input for service improvement, in particular how service providers can gain access to a range of assessment instruments in relation to person-centred care.

## Methods

### Design and data collection

We carried out a retrospective document analysis of the development of the Range of instruments. That is, we conducted an extensive review of relevant documents at three levels, i.e. 1) national documents such as policy papers and governmental reports, 2) documents and reports from the VGN and minutes from the meetings of the expert committee who assessed the instruments, and 3) correspondence between the committee and developers, and the forms used in the assessment process for each instrument.

### Analysis

Two independent researchers (i.e., who had no affiliation with the development of the Range of instruments) first read all the documents to familiarise themselves with the content. Next, all extracts identified as being relevant to the development process of the Range of instruments were inductively coded using a thematic analytical approach [31] by one researcher with the support of ATLAS.ti software [32]. In accordance with the guidelines, 20 percent of the documents were independently coded by a second researcher) to ensure the reliability and validity of the coding [33]. Any discrepancies in the initial coding were discussed among three researchers until a consensus was reached. The final codes were subsequently categorised into overarching themes, which were organised into phases. The authors jointly provided descriptions of these timeframes in the results section based on the codes.

## Results

**Fig. 1:**
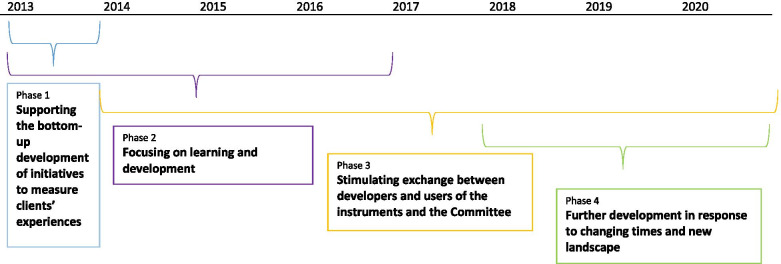
Timeline

All of the categories identified through the data analysis were plotted on a timeline (see Fig. 1), with four main phases being identified in the process of development of the Range of instruments.

### Phase I – Supporting the bottom-up development of initiatives to assess clients’ experiences (year 1: 2013)

The predominance of the client-centredness paradigm in the care sector inspired the Dutch ID care to improve their own care and support practice concerning clients’ experiences, with both independent research organisations and individual care organisations all having now developed their own instruments to assess clients’ experiences. The VGN decided to support these bottom-up ‘private’ initiatives by professionalising such individual initiatives.

In an effort to streamline all of the individual instruments that emerged in practice, the VGN sought to develop an overview of locally developed instruments to assess clients’ experiences in residential care, alongside highlighting the importance of assessing the respective (scientific) merits of these instruments. This would enable their 171 affiliated care organisations across the Netherlands to make an informed decision, from a set of pre-selected high quality, valid instruments, about which instrument would best fit the local practice of their particular organisation. These instruments had to be capable of, on the one hand, assessing individual clients’ experiences of the care and support provided to them by a specific care organisation, and, on the other, helping to directly improve that organisation’s care and support provision (both at an individual and organisational level). Given the life-wide nature of ID care and support, VGN chose to adopt the concept of quality of life (QoL) as a framework through which to assess clients’ experiences of the quality of care and support they received. Schalock et al. [34, 35] provided an operational definition of QoL for people with ID across eight domains: personal development, self-determination, interpersonal relations, social inclusion, rights, emotional, physical, and material well-being. Hence, all instruments should encompass all of these eight QoL domains.

As a first step in compiling the Range of instruments, VGN invited care organisations for people with ID and researchers to submit the instruments that they developed an used in their local practice to assess clients’ experiences and improve the quality of care and support. Subsequently, VGN installed an independent Expert Committee on Clients’ Experiences of Instruments (hereafter referred to as the Committee) comprising three scientific experts (i.e., the authors; KA and MM took over from HN and PM as committee members in 2017) to assess the submitted instruments. The assessment criteria for admission to the Range of instruments were initially defined as: 1) the assessment of individual clients’ experiences of the quality of care and support received from a particular care organisation, in accordance with the QoL domains, 2) the possibility of eliciting suggestions for improvement at the individual client level, 3) the possibility to aggregate the data deriving from individual clients to the level of a team or entire organisation, 4) the ability to embed the instrument in clients’ individual support plans to ensure that the interventions actually produce improvements, and 5) the scientific quality of the instrument (i.e., as defined by the sufficient level of reliability and validity of the data generated by the instrument or the efforts that are undertaken to increase methodological rigour). The Committee followed an eclectic approach, i.e. instruments from a diverse array of methodologies were included, both qualitative and quantitative, provided they matched the rigour of their respective methodological schools.

Making use of individual clients’ experiences to both ascertain individual suggestions for improvement and subsequently embed these within individual support plans is a unique approach, insofar as most instruments for assessing clients’ experiences only report on the aggregated team, location or organisational level. The Committee was granted the freedom to determine its own method of working to arrive at providing independent advice to VGN board members. The relationship between the Committee and the VGN board members was clear, as stated in the meeting minutes of the Committee from June 1, 2015: “*The board adopts the advice [of the Committee], because VGN lacks the expertise.*” Furthermore, because of this independent positioning, the members of the umbrella organisation VGN could not interfere with the independence of the judgements that were to be made.

### Phase II – Focusing on learning and further development (year 1-4: 2013-2016)

Based on a full assessment of the five criteria, the Committee subsequently provided judgements that corresponded to three categories, namely: (1) the instrument is admitted unconditionally to the Range of instruments, (2) the instrument is admitted to the Range of instruments with conditions attached (after having made specifications to the development), and (3) the instrument is not admitted to the Range of instruments. The Committee’s evaluation was explicitly aimed at stimulating further development of the submitted instruments. By providing advice on how to improve the reliability and validity of the instruments, the Committee strived to contribute to the “*young academic history in ID care*.” *[Appendix 2, procedure and rules Committee on Clients’ Experiences of Instruments, December 10, 2013].*

By the beginning of 2013, an overview of nine instruments was published in a digital newsletter from VGN in order to help the Dutch ID care make an informed decision regarding which instrument to apply in their care organisation. Later that year, VGN issued a second call for instruments, which resulted in an additional six instruments being submitted. At this stage, the Committee itself broadened the scope of the original assignment posed by VGN to assess instruments. Although VGN initially stated that it did not want to impose restrictions on the size of the Range of instruments, the Committee now recommended limiting the number of instruments in the Range of instruments in an attempt to establish convergence between instruments. Although this would reduce the number of instruments included in the Range of instruments, the Committee’s rationale was that the remaining instruments would be more widely applied, which, in turn, would engender an actual improvement in the quality of care and support. Moreover, this approach would also assure the developing organisation that the instrument was sufficient, and that it was worthwhile to continue to invest in the maintenance and further development of the instrument. Instruments were perceived as adding value if they were targeted at a specific client group that the previous Range of instruments were deemed to be unsuitable for, such as those with PMID.

The assessment procedure and criteria for evaluating (candidate) instruments for the Range of instruments were the outcome of an organic, inductive process based on the experiences that the Committee had gained along the way. As the Committee stated in 2014: *“Working with quality instruments from the field is in itself just as much a development process as it is for the instruments. So we learn on the basis of experiences and feedback.” [Conversation note Committee, January 13, 2014].* Hence, in 2014, based on an identified gap in the Range of instruments concerning the client-base, the Committee asked VGN to issue a call only for those instruments that sought to gain insight into the experiences of clients with PIMD.

This call resulted in the submission of five instruments, of which one was subsequently added to the Range of instruments. The ongoing development of the assessment process resulted in the Committee retrospectively questioning some of the relatively positive judgments they had made about instruments from the initial batch of submissions. Their recommendation to re-evaluate the complete composition of the Range of instruments was taken up by VGN, with the first comprehensive reassessment taking place in September 2016. After this reassessment, a total of seven instruments remained in the Range of instruments, of which four were unconditionally included and three were conditionally included. In addition, one instrument was rejected, one decision was postponed, and two instruments were withdrawn by the developers. Once again, the Committee considered the further development of the instruments to be of the utmost importance, and, to this end, they provided submitters with advice concerning the following: 1) making outcomes more suitable for actually improving care and support at the level of the individual client (e.g., no anonymous data collection), 2) the methodological properties of the instrument (i.e., reliability and validity), and 3) actual use of the instrument (e.g., provision of a manual, information about the training required for users of the instrument, indication of the time frame needed to administer the instrument).

### Phase III – Stimulating exchange between developers and users of the instruments and the Committee (year 2-8: 2014-2020)

Initially, in order to preserve its independent position, the Committee opted not to communicate with the developers of the submitted instruments, with all correspondence instead being handled by the VGN’s office. In retrospect, the strict position adopted by the Committee in the first two years can be summarised as follows: *“The relationship between the committee and the field is clear: there is none.” [Report meeting Committee, January 20, 2014].* However, all the involved parties came to see that direct dialogue was useful. The first so-called platform meeting in which both the developers of the instruments and the Committee were present was initiated in 2014 by VGN. This meeting was motivated by the Committee’s recommendation to limit the size of the Range of instruments. *“In order to actually increase the quality of the Range of instruments, which may mean that a certain degree of convergence takes place, the proposal is to set up a Platform to facilitate exchanges between submitters and developers of the instruments.” [Note from the VGN board meeting, July 4, 2013].* Due to the success of the inaugural meeting, it was decided that this meeting should be held every other year for the express purpose of stimulating exchange between developers and (future) submitters of instruments in order to explore potential collaborations. The meetings also provided a platform for discussing generic issues or gaps, such as ‘the aggregation of qualitative data derived from interviews with clients about their experiences in a robust albeit affordable way’.

While the platform meetings primarily focused on knowledge exchange and stimulating collaboration between current and future submitters, in March 2016, the first meeting was organised for both the developers and users of the instruments included in the Range of instruments and the Committee. The aim of this meeting was not to assess the instruments, but rather to have an open exchange of perspectives, focused on user experiences with the instruments, methodological issues and further development of the instruments. For each instrument, the Committee had a conversation with the instrument developer(s), a manager from (one of) the care organisations that used the instrument, support staff who administered the instrument and one or two clients who completed the instrument. Based on the positive evaluation of these meetings, the developers, users and the Committee have met every other year since.

The exchanges with ID care practitioners provided the Committee with valuable input into (refining) the assessment criteria. Based on these conversations, the Committee came to recognise the importance of the long-term availability of instruments in the Range of instruments, given the level of (financial) investment on the behalf of care organisations to implement any of the instruments selected from the Range of instruments. Hence, the Committee encouraged developers whose instruments were deemed to be vulnerable apropos long-term availability and sustainability to make a business case for their continued inclusion, before, on the basis of these outcomes, deciding whether to continue with the development of the instrument. The Committee’s proposal to add a new assessment criterion related to long-term availability and sustainability was adopted by VGN. The accompanying assessment criterion was defined as follows: “*There are guarantees from the developer for continuity in terms of survival and further development (i.e., long-term availability).*” *[Final Report Committee on Clients’ Experiences Instruments, September 2016].*

Furthermore, based on feedback from the ID care practitioners, the Committee subsequently reviewed the fit between the instrument(s) and the organisations that (will) use it. The Committee paid special attention to the contextual requirements involved in the preparation and actual use of an instrument (e.g. costs and time incurred by the care organisation in implementing it). The accompanying assessment criterion was defined as follows: *“It is made explicit under which circumstances an instrument is useful, and under which conditions it will be adequate.” [Final Report Committee on Clients’ Experiences Instruments, September 2016].*

Following the call for instruments suitable for collecting experiences from clients with PIMD in 2014, the Committee subsequently held an expert meeting in 2017 to jointly discuss assessing the experiences of clients with severe disabilities. Whereas originally the Committee applied the criterion that clients’ experiences should be assessed by the clients themselves, there was agreement regarding the difficulties that this criterion posed for people with PIMD. However, an inherent problem with proxy-assessment is the risk associated with misinterpreting the (non-verbal) communication of clients, which, in turn, can potentially lead to both inadequate assessments of clients’ experiences of care and support and adverse alterations being made to care practices. In the expert meeting, the attendants jointly decided to add a criterion for the assessment of instruments for clients who have difficulties with verbal communication: “*data will be collected in administering the instrument in at least two persons, who are involved in the client’s care and support from different perspectives.” [Report expert meeting PIMD, April 26, 2017].* Moreover, since the attendants considered that these instruments were not only suitable for people with PIMD, the target group for these instruments was also broadened to include *“clients who are incapable of verbally expressing themselves in such a way that people in their surroundings can ‘understand’ them, including those who are familiar with the client.” [Report expert meeting PIMD, April 26, 2017].*

### Phase IV – Further development in response to changing times and new landscape (year 6-8: 2018-2020)

The current Range of instruments consists of 11 instruments^2^ (see Table 1 for the key characteristics of these instruments).

**Table 1:** Key characteristics of the instruments included in the Range of instruments 2020-2022 (*N*=11)

	N	Description of instrument
Instrument’s adequacy for target groups		
Mild ID Mild ID (youth) Moderate ID Severe ID PIMD Proxy assessment Acquired brain injury Sensory impairment Physical impairment	7 9 8 8 2 8 7 5 5	
Instrument developers		
External agency	10	Care organisation 1 should be in the same row as External agency 10
Care organisation	1	
Method of data collection		
1. Dit vind ik ervan – Ik vertel [This is what I think – I tell]		Topic list with 10 subjects (feelings, body, family, circle of friends, help, inclusion, home, activities, choice/influence, feeling safe) that can be related to the quality of life dimensions of Schalock and Verdugo, investigative, appreciative interview of the client with the mentor as proxies to explore what is important to the client and how this has been experienced, a qualitative approach.
2. Dit vind ik ervan – Ik toon (PIMD target group ) [This is what I think – I show]		Topic list with same topics as in DVIE-Ik Vertel, observation/film recordings, a qualitative approach, dialogue based on film recordings of involved relatives and care professionals with clients who can only communicate non-verbally.
3. Ben ik tevreden [Am I satisfied]		Topic list with 8 topics (physical well-being, psychological well-being, personal development, self-determination, interpersonal relationships, participation, material well-being, rights) and a module for work and daytime activities, topics are closely related to the Schalock and Verdugo dimensions and are elaborated in a set of detailed guiding questions.
4. Ben ik tevreden (PIMD target group ) [Am I satisfied]		Observation list based on the same topics as in Ben ik tevreden, the topics (supported by a set of guiding observations) are applied in a dialogue between representative and mentor of the client (as proxies) who assess body language and the personal experiences of the client and determine the scores and actions.
5. C-toets OBC [C-test OBC]		Questionnaire specifically designed to elicit experiences of the care and treatment of children, youths and adolescents with mild ID or severe behavioural problems, both for the youths and their parents with fixed categories (mentors, goals and treatment, information, the group, rules; inpatient and outpatient version), the possibility to add several questions (e.g. safety, leisure, school, work, daytime activities) and a few open questions about what can be improved. Youths and parents participated in the redesigning of the questionnaire.
6. Clientervaringsonderzoek [Client experience survey]		Questionnaire, 8 quality of life dimensions (Schalock and Verdugo), 6 scales (mentors, support, living environment, daytime activities, leisure and contacts, care/support plan), instrument is based on 20 fixed questions and offers the possibility to add questions which can be tailored in a personalised way.
7. Mijn mening [My opinion]		Questionnaire, 4 categories (individual control, treatment, leisure & room furnishing, group climate & atmosphere), questions are checked for literacy, instrument is available as a web app.
8. Onze cliënten aan het woord [Our clients have their say]		Questionnaire and open questions, 7 modules (mentoring, living, daytime activities, leisure time, medical care, care/support plan and participation/complaints), 4 quality of life questions. Specific questions about what can be improved and what needs to be cherished are included.
9. Quality Cube		Questionnaire and open questions, 8 quality of life dimensions (Schalock and Verdugo), 4 enabling dimensions based on Inspectorate’s quality indicators, 5 service-related dimensions based on SERVQUAL model. Quality Improvement Charts are reported at the team level.
10. POS (Personal Outcome Scale )		Interviews by an independent interviewer guided by a questionnaire, which is based on Schalock and Verdugo’s dimensions (personal development, self-determination, interpersonal relations, social inclusion, rights, emotional, physical, and material well-being), the POS is applied in many countries worldwide.
11. Cliënten over kwaliteit [Clients about quality]		Questionnaire/focus group meeting/mirror meeting, 3 questionnaires (living, daytime activities, ambulatory care) with 23 fixed questions and 4 open questions, followed by focus group meetings (6-12 persons with involvement of client board) and mirror meetings (6-10 clients and 6-10 employees).

Since the initiation of the Range of instruments, the Committee has provided (re) assessments of what instruments are to be included in the Range for three to four-year periods at a time (i.e., 2013-2016; 2017-2019). The criteria on which these (re) assessments are based are described in Table 2. Within these three-year phases, the Committee annually assesses whether the instrument developers need to undertake steps towards further development of their instrument, based on feedback from the developers themselves. On the eve of the third assessment period (2020-2022), the Committee reflected on both the assessment criteria and some of the original, guiding principles of the Range of instruments.

**Table 2:** Range of instruments for measuring clients’ experiences - assessment criteria 2020-2022

Criterion	Aim	Year of inclusion
1. The instrument yields information at the level of the individual client	Giving voice to individual clients	2012
2. The instrument provides insight into the experiences and concrete suggestions for improvement of individual clients, and is specifically tailored to people with PIMD; explained under (d): a) The person himself is speaking, as opposed to a proxy; b) The instrument does not only record the current situation, but also explicitly affords the possibility to make suggestions for improvement in the individual client’s life; c) In such a way that the person’s own frame of reference is recognisable; d) In order to collect data on people with PIMD, the instrument needs to be administered by at least two people, who are involved in the client’s care from different perspectives (e.g. a relative and a member of the support staff).	Ensuring that suggestions for improvement can be dealt with at an individual level	2012 (a – c); 2017 (d); to avoid misinterpretation of the (non-verbal) communication of clients with PIMD, which can possibly lead to the inadequate assessment of clients’ experiences of care and undesirable alterations being made to care practices.
3. Use of the instrument is embedded in the care plan cycle (i.e., methodical discussion of the individual care plan)	Aligning the suggestions for improvement with the work processes of the care organisation, and ensuring that actions are carried out	2012
4. Data can be aggregated (anonymously) to different levels (team, location, organisation)	Enabling benchmarking at the team level and over time	2012
5. It is explicitly stated under which circumstances an instrument is useful, and under which conditions it will be fully appreciated	Ensuring that the necessary contextual circumstances to apply the instrument are met, so that the impact of the instrument is most effective	2016; based on conversations with ID care practice
6. Instrument developer(s) can guarantee continuity in availability and the (further) development of the instrument	Ensuring long-term availability	2016; based on conversations with ID care practitioners
7. The instrument yields reliable assessments	Ensuring the methodological soundness of the instrument	2012
8. The instrument is valid (face validity, construct validity, criterion validity)	Ensuring the methodological soundness of the instrument	2012

In an attempt to further develop the psychometric properties of the instruments, and thereby increase the methodological soundness of the assessment of clients’ experiences, the Committee divided the assessment of the instruments’ reliability and validity into two separate criteria. Next, whereas in the first assessment periods the developers also reported on the validity and reliability of their respective instruments, instrument developers will now be asked to provide a specific in-depth report on the psychometric properties of their instrument, which will then be assessed by external methodological experts to guarantee a fresh perspective.

In every consecutive assessment period, both instruments which are already included in the Range of instruments and those that are newly submitted will be assessed based on the same criteria. Given that the assessment process is an iterative and evolving process, the reassessment of instruments included in the initial years proved to be a challenging process for the Committee at times. Next, newly submitted instruments are required to offer added value to those instruments already included in the Range of instruments, with respect to the targeted client-base and/or provide a distinctive mode of data collection (the latter aspect was added in 2019). However, the Committee thought that this check for added value was “unfair” to those instruments included in the initial years.

Whilst acknowledging the relevance of Schalock’s QoL domains for assessing clients’ experiences of care and support, the Committee sought to broaden this guiding concept to fit the present juncture: *“In recent years other concepts have emerged, such as ‘positive health’* *[*36*]. In addition, in aiming to stimulate clients’ participation in society, concepts such as autonomy and independence have gained relevance.” [Meta-advice Committee on Clients’ Experiences Instruments, October 2018].* In line with this observation, the Committee advised VGN in 2018 to “*offer space for other concepts than Schalock’s QoL domains, for example positive health and autonomy.” [Meta-advice Committee on Clients’ Experiences Instruments, October 2018].*

Next, from the outset, the Range of instruments was intended to include instruments capable of assessing the experiences of clients in residential ID care. However, the Committee also observed that there was both interest in using instruments from the Range of instruments and a desire to submit new instruments from adjacent fields of (ID) care and support, such as care organisations providing community-support or day-care activities for people with ID and/or psychiatric care. Driven by this interest in gaining insight into the perspectives of clients themselves, in 2018 the Committee advised VGN to broaden the scope of the Range of instruments from only residential care to day-care activities and community-support for people with ID. Ultimately, VGN decided to maintain the focus on residential ID care and support*.* Broadening the scope was deemed to be premature in light of the fact that the Range of instruments is an integral part of a process that seeks to optimise the quality of care for long-term ID, which runs until 2022 and which is covered by the Long-term Care Act. Furthermore, Dutch municipalities are also responsible for the provision of day-care activities and community-support under different legislation (the Social Care Act). Therefore, VGN wishes to explore their views on the assessment of clients’ experiences through the Association of Dutch Municipalities.

## Discussion

The aim of the current study was to provide a unique bottom-up, exhaustive account of the process of developing a Range of instruments used to assess clients’ experiences in such a way that contributes to an improvement in the quality of care and support, which aligns with the increased attention being paid to the inclusion of experiential knowledge within ID care and support [37, 38]. In this respect, the Netherlands and Dutch ID care serve as a case study into how we approached the task of making clients’ experiences explicit in a systematic manner, so that they could be used as input for service improvement, in particular how service providers can gain access to a range of assessment instruments in relation to person-centred care.

The assessment of clients’ experiences of the quality of care and support they have received, through the use of instruments deemed to be suitable by an independent committee, has contributed to clients’ individual perspectives being firmly embedded in the care process. Through manifold assessment criteria, the Committee has explicitly underlined the need for instruments to correspond to specific work processes of individual care organisations. Only in this way can instrument outcomes genuinely contribute to improvements in the actual care and support provided to individual clients. This is important, as it has been noted previously that even when there is a broad consensus regarding the principles of client-centeredness, this in and of itself does not necessarily translate into a single model of client-centred care [39]. Moreover, by ensuring that instruments are included in methodical discussions of clients’ individual care plans, outcomes will come to occupy a natural place in the improvement cycle at the level of the individual client, team and the entire organisation. In this way, instruments for assessing clients’ experiences can thus be said to contribute to learning and quality improvement [40]. It should be noted however, that although the Range of instruments fits in with the increased attention paid to experiential knowledge in ID care and support, there was nevertheless resistance from healthcare insurers when the Committee first introduced their ideas for the Range of instruments. That is to say, this Range of instruments did not match their firm beliefs pertaining to benchmarking, accountability and the use of quantitative data for the purposes of comparability. Now that the idea of the Range of instruments has descended, it has become more broadly accepted.

The fact that the VGN initiated the Range of instruments can be viewed as an enabling factor for the successful development and subsequent use of instruments in this Range. In its capacity as the representative for ID care organisations, VGN has the support of managers of care organisations, who themselves play a pivotal role in knowledge and innovation processes, namely in terms of both driving the choice for one instrument over another and engendering an organisational culture predicated on quality improvement rather than accountability [41]. In other fields of care, such as the field of integrated care, the fact that development is initiated at the local level has also been found to be an important determinant of success [42]. It has even been stated that when such initiatives fail, this is often due to the fact that they are invariably top-down approaches [43]. However, the Committee sees notable variation in the level of intrinsic motivation held by care organisations to truly improve care and support based on clients’ experiences and preferences. While there are certainly highly motivated organisations, there are also several organisations that merely make use of an instrument at the ‘last minute’ just so they can say that they have done so. It would be interesting for future research to explore the actual uptake and implementation of the instruments and their follow-up action points within ID care organisations. In this respect, one important aspect, amongst others, would be to collect information on the psychometric properties of the instruments. During the bottom-up approach, the importance placed upon this information shifted over time. That is to say, whereas a clear description of the content of the instruments was deemed to be of paramount importance for the Committee during the initial phase, with less emphasis being placed on the psychometric properties of the instruments, more detailed information about the reliability and validity of the instruments was eventually required as the process progressed. In line with the adopted bottom-up approach, it can be concluded that the psychometric properties of some instruments are currently better examined than others, although, based on the unpublished data presented by the developers, most can be considered to be acceptable. Nevertheless, to further enhance the value of the instruments, the Committee stresses the importance of instrument developers both exploring and demonstrating the psychometric properties of their instruments. This paper focused on the development of suitable instruments through which to assess the experiences of clients receiving ID care and support. Although this is an imperative first step, it is only one part of carrying out an effective evaluation process. Future research should therefore also include associated issues, such as the level of training and support provided to the personnel who carry out the assessments, the link between assessed needs and the outcomes of the actions taken to meet those needs, and the managerial processes required to monitor and refine the use of assessments within the services.

Undoubtedly, data on clients’ experiences of the care and support they receive is invaluable for other relevant parties, including healthcare purchasers and policy-makers. Although their primary interest in care provision is financially driven, it is nevertheless essential to engender a cultural shift towards learning and development. Next, using individual client information at the aggregated level also highlights the challenges involved in combining huge amounts of qualitative data. In particular, the aggregation of qualitative data that has been collected non-anonymously represents a recurring issue for the Committee, as well as constituting a notable challenge for both instrument developers and care organisations. In this respect, it would be important to transcend national boundaries, insofar as issues such as aggregation of qualitative data are not only an issue in the Netherlands, of course. Outside the Netherlands, there are pre-existing assessment systems for doing this, such as the POM [28]. Both the developers of the Dutch instruments as well as the VGN and ID care organizations can learn from such instruments and their attempts to, for example, collect data at a national, or even international, level. In addition to transcending national boundaries, it might also be interesting to transcend this specific care sector (i.e., ID care) and to explore whether the instruments included in the Range could also be applied in other sectors, such as care for the elderly (i.e., long-term care). It is important to mention here that some of the Dutch instruments in the Range of Instruments have also been used and tested outside the Netherlands, such as the Personal Outcome Scale [44], whereas others originally developed in a bottom-up manner within a single ID care organisation before subsequently being implemented within other Dutch ID care organisations. Hence, the current stage of the instruments varies greatly, and, hence, it is important to follow-up on how the instruments develop in the coming years.

The development process of the Range of instruments is characterised by its incremental nature, continuous evaluation and strong practice orientation, as well as the use of expert knowledge and scientific and methodological rigour. Indeed, the assessment criteria for including instruments in the Range of instruments, the content of the Range of instruments, as well as the communication between the Committee and the field of ID care have all evolved over the years. The scope for flexibility and adjustments that VGN offered to the Committee, both in terms of the instruments and with respect to their own development processes, made it possible for both parties to collaboratively learn via the process of engagement. For example, the Committee adjusted its assessment criteria in response to feedback from the field, while instrument developers used the Committee’s assessments to improve their instruments. Continuous evaluation has been another important characteristic of the ever evolving process concerning what instruments are included in the Range of instruments. Both the VGN and the Committee attach great importance to knowing which instruments are being used, as well as if the instruments are actually working in the way they were designed to by developers, with respect to, among other things, time, cost, reporting and the target group [45]. This has repeatedly been found to be an important quality in driving innovative processes [46], and the development process of the Range can certainly be classified as such. For the purpose of the current study, we carried out a retrospective document analysis of the development of the Range of instruments. Although exploring which instrument performs ‘best’ was ultimately beyond the scope of this paper, it would be interesting for future research to interview the developers of the instruments as well as ID care organisations and their clients who use the instruments, in order to explore their experiences with using the instruments, to identify the scale at which the different instruments are being used, and to explore future development opportunities (e.g., with scientific institutes).

The Range of instruments is a bottom-up approach that serves as a direct alternative to the top-down (imposed) CQ-index, which is framed by the field of ID care and support as a quantitative and standardised instrument that assesses patients’ experiences in healthcare at an organisational and team level with a particular focus on accountability and benchmarking. VGN and the Committee succeeded in establishing an alternative to this approach by compiling a range of high-quality instruments that do justice to the variety of clients in ID care, assessing individual clients’ experiences with a specific focus on collaboratively improving the quality of ID care and support by means of learning and development. These desired characteristics are not without their own issues however, particularly as it concerns the difficulties involved in aggregating qualitative data and the wealth of information that results from combining all of the available data, which have hitherto not been resolved. Furthermore, in order to include the entire field of ID care and support and truly take the client as the starting point as opposed to the facility where the care and support is provided, the Committee is seeking to broaden the Range of instruments into adjacent care sectors, such as community-support in all its various forms.

Currently, the Range of instruments consists of 11 instruments, which all meet the assessment criteria developed by the Committee. Evidently, those instruments that failed to meet the criteria are not included in the Range of instruments. In this respect, it is important to note here that methodological issues were not the only reason for not being included in the Range of instruments. Rather, instruments could also be rejected or postponed on the grounds of ethical concerns. An example of this would be commercial parties who developed an instrument comparable to the CQ index with the primary aim of providing opportunities for benchmarking and accountability.

To conclude, the underlying approach adopted in the development of the Range of instruments facilitates client-centeredness for the express purpose of improving the quality of care and support, and, as such, can be effectively applied across a range of care, regional and national contexts. Naturally, the development process of the Range of instruments is influenced by the specific national context in which it transpires, such as, for example, in terms of the general health, long-term care and social care system in the Netherlands, not to mention the fact that the living conditions of people with ID in the Netherlands differs markedly from those in other Western countries, inasmuch as a large percentage of these clients live in institutions [47]. However, while the key factors which enabled the Dutch policy objective of client-centeredness to actually be successfully translated into outcomes in practice – its incremental nature, continuous evaluation and strong practice orientation – at least to the best of our knowledge, are highly innovative, they can nevertheless be applied in many other countries seeking to develop a high quality of care and support for people with ID. However, in an era in which uniformity, standardisation and benchmarking are paramount, it will require considerable effort and persistence on the behalf of all stakeholders involved to faithfully pursue this path. Pursuing this path, however, is of vital importance for implementing client-centred care. That is to say, through the use of an instrument that is included in the Range, people with ID can directly describe what they want from their lives. Consequently, ID care organisations can tailor the services they provide to facilitate those outcomes, providing benefits both at the individual level, for example by using an instrument to both inform a truly person-centred plan and to track the progress in executing that plan, and at an aggregate level, by, amongst other things, collecting data to both examine the impact of their support and recognise effective services, while also being able to highlight areas where additional work is required.

## Conclusions

Whilst Dutch ID care deemed that the assessment of clients’ experiences of care and support was an appropriate method through which to improve the quality of ID care and support, the prevailing standardised instruments that focused on quantitative measurement were not considered to be suitable for the specific needs of ID care and support. In this paper, a unique process was described, in which a specific field of care sought to implement the national top-down policy objective of client-centred care and support (i.e., via VGN with 171 affiliated ID care organisations) through adopting a bottom-up approach. Rather than focusing on benchmarking and accountability, the ID care sector explicitly aimed to jointly improve the quality of care and support, in such a way that foregrounded clients’ experiences of care and support and their suggestions for improvement. The subsequent development of the Range of instruments is an ongoing process, as evidenced by the current interest from adjacent fields of care to either submit instruments to, or use instruments from, the Range of instruments.

## Data Availability

The datasets generated and/or analysed in the present study are not publicly available due to the fact that the data is traceable to individuals, which raises the possibility of individual privacy being compromised. However, the data is available from the corresponding author upon reasonable request.

## Abbreviations

ID: intellectual disabilities
PIMD: Profound intellectual and multiple disabilities
QoL: Quality of life
CQ-index: Consumer Quality Index
VGN (Dutch abbreviation): Dutch Association of Healthcare Providers for People with Disabilities

## Footnotes

1 There are differences in terms of how the care and support for people with intellectual disabilities is classified in various policy sectors. In this paper, long-term care is used, but in other countries it can be part of social care and social security legislation.

2 The following instruments are included in the Range of instruments: 1) Dit vind ik ervan – Ik vertel [This is what I think – I tell]; 2) Dit vind ik ervan – Ik toon (PIMD target group) [This is what I think – I show]; 3) Ben ik tevreden [Am I satisfied]; 4) Ben ik tevreden (PIMD target group) [Am I satisfied]; 5) C-toets OBC [C-test OBC]; 6) Clientervaringsonderzoek [Client experience survey]; 7) Mijn mening [My opinion]; 8) Onze cliënten aan het woord [Our clients have their say]; 9) Quality Cube; 10) POS (Personal Outcome Scale); 11) Cliënten over kwaliteit [Clients about quality]
